# Oxidative Stress Markers and Modified Model for End-Stage Liver Disease Are Associated with Outcomes in Patients with Advanced Heart Failure Receiving Bridged Therapy with Continuous-Flow Left Ventricular Assist Devices

**DOI:** 10.3390/antiox10111813

**Published:** 2021-11-15

**Authors:** Bożena Szyguła-Jurkiewicz, Wioletta Szczurek-Wasilewicz, Mariusz Gąsior, Izabela Copik, Justyna Małyszek-Tumidajewicz, Michał Skrzypek, Ewa Romuk, Michał Zembala, Marian Zembala, Piotr Przybyłowski

**Affiliations:** 1 3rd Department of Cardiology, School of Medical Sciences in Zabrze, Medical University of Silesia, 40-055 Katowice, Poland; bjurkiewicz@sum.edu.pl (B.S.-J.); mgasior@op.pl (M.G.); 2 Silesian Center for Heart Diseases in Zabrze, 41-800 Zabrze, Poland; i.copik@sccs.pl (I.C.); jmalyszek@sccs.pl (J.M.-T.); mzembala.jr@sccs.pl (M.Z.); mzembala@sccs.pl (M.Z.); pprzybylowski@sccs.pl (P.P.); 3 Department of Cardiac, Vascular and Endovascular Surgery and Transplantology in Zabrze, 41-800 Zabrze, Poland; 4 Department of Biostatistics, School of Public Health in Bytom, Medical University of Silesia in Katowice, 40-055 Katowice, Poland; mskrzypek@sum.edu.pl; 5 Department of Biochemistry, School of Medical Sciences in Zabrze, Medical University of Silesia in Katowice, 40-055 Katowice, Poland; eromuk@sum.edu.pl

**Keywords:** oxidative stress, modified model for end-stage liver disease, heart failure, left ventricular assist device

## Abstract

Left ventricular assist device (LVAD) is well established as an alternative treatment for end-stage heart failure (HF) patients. The aim of the study was to determine the prognostic value of oxidative stress markers and the modified Model for End-Stage Liver Disease (modMELD) in patients receiving bridged therapy with continuous-flow LVAD. We prospectively analyzed 36 end-stage HF patients who received LVAD therapy between 2015 and 2018. The total antioxidant capacity (TAC) and total oxidant status (TOS) were measured by the methods described by Erel. The oxidative stress index (OSI) was defined as the ratio of the TOS to TAC levels. The modMELD scores were calculated based on the serum bilirubin, creatinine, and albumin levels. The patients’ median age was 58 (50–63.0) years. During the 1.5-years follow-up, a major adverse cardiac event—MACE (death, stroke, or pump thrombosis) was observed in 17 patients (47.2%). The area under the receiver operating characteristics curves (AUCs) indicated a good prognostic power of TAC (AUC 0.7183 (0.5417–0.8948)), TOS (AUC 0.9149 (0.8205–0.9298)), OSI (AUC 0.9628 (0.9030–0.9821)), and modMELD (AUC 0.87 (0.7494–0.9905)) to predict a MACE. Oxidative stress markers serum concentrations, as well as the modMELD score, allow the identification of patients with a risk of MACE.

## 1. Introduction

Despite continuous advances in medicine, heart transplantation (HT) is the most effective treatment for end-stage heart failure (HF) patients who are refractory to medical therapy. However, because of a limited number of suitable donor organs available, the mortality on waiting lists remains high [1,2]. Over the last decades, implantable left ventricular assist devices (LVADs) have become an alternative treatment for advanced HF patients, and their use as a bridge to transplant has been widely increasing [3,4]. The shift away from pulsatile flow technology and toward continuous technology (the second and third-generation LVADs) is associated with the improvement of outcomes. The continuous-flow LVADs are smaller in size and require less surgical dissection and less time for implantation than the pulsatile LVADs. Furthermore, favorable outcomes after an LVAD implantation still depend on patient selection, perioperative risk stratification, and long-term clinical management. Therefore, noninvasive sensitive tools are necessary to select patients with worse clinical outcomes after the procedure [5]. 

Considering the underlying pathophysiological mechanisms of HF as well as the possible changes in myocardial oxidative stress in patients with the support of an LVAD, we aimed to determine the prognostic value of oxidative stress markers in patients receiving bridged therapy with continuous-flow LVADs. Furthermore, we sought to analyse the utility of the modified Model for End-Stage Liver Disease (modMELD) scores in the assessment of outcomes in the analyzed group of patients. 

## 2. Materials and Methods

### 2.1. Study Population and Data Collection

The study is a prospective analysis of 36 end-stage HF patients (Interagency Registry for Mechanically Assisted Circulatory Support [INTERMACS] 2 and 3, NYHA [New York Heart Association] IV) who were hospitalized in the Cardiology Department and received LVAD therapy as a bridge to HT between 2015 and 2018. All of the included patients were eligible for LVAD implantation in accordance with European and American guidelines [3,6]. The patients were supported by continuous-flow devices—the axial-flow left ventricular assist systems (HeartMate III, St. Jude Medical, Inc., St. Paul, MN, USA) or the magnetically levitated, centrifugal flow pump (the HeartWare HVAD (Medtronic, Inc., Framingham, MA, USA). All patients were considered transplant candidates at the time of the LVAD implantation. 

Data on clinical characteristics and medical treatment were collected by interviewing the patients, as well as reviewing the electronic records. Preoperative laboratory values were defined as the last available set of results prior to the LVAD implantation. Before the LVAD implantation, a panel of laboratory tests were performed in all patients, along with transthoracic echocardiography with the evaluation of right ventricular (RV) function based on the following parameters: tricuspid annular plane systolic excursion (TAPSE), RV dimensions, qualitative assessment of RV systolic function, and right ventricular systolic pressure (RVSP). After device implantation, the routine management included early extubation and, depending on clinical conditions, mobilization. After the discharge, all patients underwent cardiac rehabilitation and were then periodically evaluated at our dedicated outpatient clinic.

The composite end-point of the study included death, cerebral event, or pump thrombosis during a 1.5-year follow-up. A cerebral event included an ischemic or hemorrhagic stroke. The study protocol conforms to the Declaration of Helsinki, and it was approved by the appropriate ethics review board.

### 2.2. Pharmacological Treatment

All of the included patients received optimal HF therapy for at least three months before entering the study. [6]. In addition, after the LVAD implantation, patients were initially treated with a standard anticoagulant regimen consisting of unfractionated or low molecular weight heparin in the early postoperative period, followed by warfarin (with the international normalized ratio [INR] between 2 and 3). On the first postoperative day, antiplatelet treatment with aspirin or clopidogrel (used only in the case of resistance to aspirin or other indications for this drug) was started at a dose of 75 mg/day. 

### 2.3. Laboratory Measurements

Peripheral blood was collected twice on admission and 6 months after inclusion in the study. Blood samples were obtained after 12 h or more of fasting. Standard laboratory tests were measured as soon as possible. The hematologic parameters of patients have been analyzed using automated blood cell counters (Sysmex XS1000i and XE2100, Sysmex Corporation, Kobe, Japan). Biochemical parameters such as creatinine, bilirubin, cholesterol and albumin plasma concentrations were determined with a COBAS Integra 800 analyzer (Roche Instrument Center AG, Rotkreuz, Switzerland). The N-terminal pro-brain natriuretic peptide (NT-proBNP) plasma concentrations were measured with a commercially available kit (Roche Diagnostics, Mannheim, Germany) on an Elecsys 2010 analyzer with the analytical sensitivity of <5 pg/mL).

The fibrinogen plasma concentrations were measured using the STA Compact analyzer (Roche). A highly sensitive latex-based immunoassay was used to detect the C-reactive protein (CRP) plasma concentrations with the Cobas Integra 70 analyzer (Roche Diagnostics, Ltd., Rotkreuz, Switzerland). The CRP levels were determined with a typical detection limit of 0.0175 mg/dL. 

For oxidative–antioxidative parameters measurements, a 5-mL blood sample was additionally collected into a plastic tube containing potassium EDTA. Serum samples were centrifuged at 2000× *g* for 10 min and stored in aliquots at −80 °C pending a biochemical examination. The concentration of malondialdehyde (MDA) concentration was determined according to the method described by Ohkawa et al. [7]. The total oxidant status (TOS) and total antioxidant capacity (TAC) were determined using the methods developed by Erel [8,9]. 

### 2.4. Scales

Based on the obtained data, the modMELD score and oxidative stress index (OSI) were calculated using the appropriate formulas:

− OSI = (TOS, μmol/L)/(TAC, mmol/L) [10];
− modMELD = 1.12 × (ln 1) + 0.378 × (ln total bilirubin, in mg/dL) + 0.957 × (ln creatinine, in mg/dL) + 0.643; for an albumin concentration ≥4.1 g/dL;
− modMELD = 1.12 × (ln [1 + 4.1—albumin, g/dL)]) + 0.378 × (ln total bilirubin, in mg/dL) + 0.957 × (ln creatinine, in mg/dL) + 0.643, for an albumin concentration <4.1 g/dL [11].

For the calculation of the modMELD score, the lower limit for creatinine, bilirubin, and albumin concentrations was set to 1.0, and the upper limit for the creatinine concentration was set to 4.0 mg/dL.

### 2.5. Statistical Analysis

The statistical analysis was calculated with SAS software, version 9.4 (SAS Institute Inc., Cary, NC, USA). Continuous variables are expressed as the median (upper and lower quartiles) for normal distribution data or mean ± standard deviation (SD) for nonnormal distribution data. Categorical variables are presented as frequency tables. The groups were compared using the Student’s *t*-test, the Mann–Whitney test, or the χ^2^ test. The discriminatory power of modMELD and oxidative stress markers for predicting composite end-point was evaluated by calculating the area under the curve (AUC) from the receiver operating characteristic (ROC) analysis. An AUC > 0.7 was considered clinically relevant [12]. The Youden criterion was used to determine the optimal cutoff value for the analyzed parameters. The utility of each parameter to determine the composite end-point was evaluated by sensitivity, specificity, the positive predictive value (PPV), the negative predictive value (NPV), and accuracy. *p* < 0.05 was considered as a statistically significant. 

## 3. Results

In total, 36 patients received an LVAD: 28 patients (77.8%) were supported by axial continuous-flow devices (Heartmate III, St. Jude Medical), while eight patients (22.2%) received centrifugal continuous-flow devices (Heartware International Inc., Framingham, MA, USA). All included patient were classified in New York Heart Association (NYHA) functional class IV and profiles 2 to 3 according to the INTERMACS classification (14 patients in INTERMACS 2 and 22 patients in INTERMACS 3). During a 1.5-year follow-up, 17 patients (47.2%) reached a composite end-point of the study (10 died, four had cerebral events, and three had pump thrombosis). 

The characteristics of the study population are summarized in Table 1. None of the implanted patients had significant bleeding, pump dysfunction requiring replacement, or right HF during a 1.5 years follow-up. 

The comparison of laboratory parameters before and 6 months after the LVAD implantation is presented in Table 2. There were significant differences in the improvement of the kidney and liver function parameters, as well as a reduction in the NT-proBNP levels within six months from the LVAD implantation, although there were no differences in the oxidative stress parameters in this respect. 

**Figure 1:**
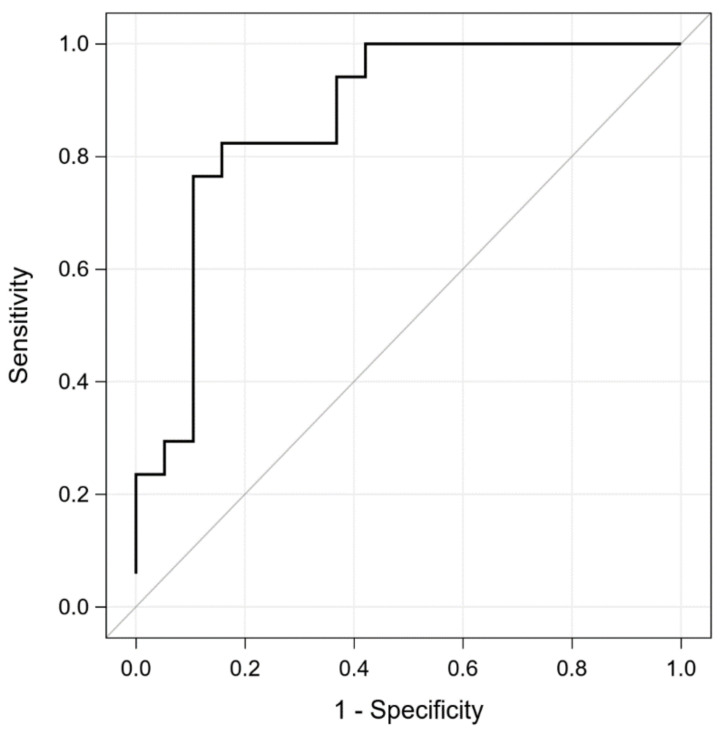
The ROC curve for modMELD score. Abbreviations: modMELD, modified Model for End-Stage Liver Disease; ROC, receiver operating characteristic.

**Figure 2:**
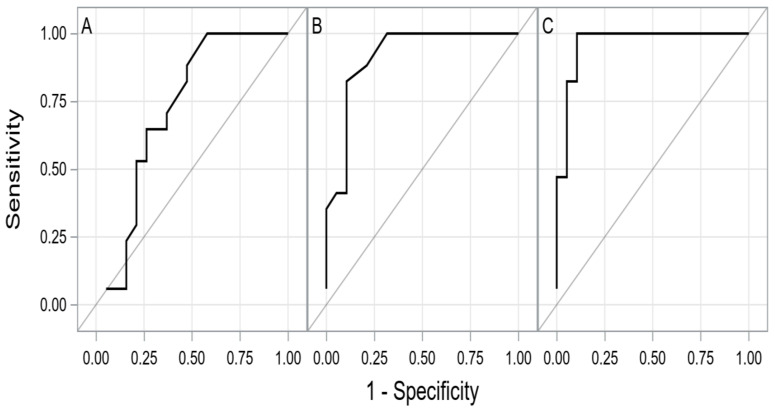
The ROC curves for TAC (**A**), TOS (**B**), OSI (**C**) levels. Abbreviations: OSI, oxidative stress index; TAC, total antioxidant capacity; TOS, total oxidant status.

The summary of the ROC curves analysis for biomarkers predicting a composite end-point during the 1.5-year follow-up is presented in Table 3. The ROC curve for modMELD is shown in Figure 1. The comparison of ROC curves for oxidative stress parameters is presented in Figure 2A–C.

## 4. Discussion

This single-center, prospective study is the first one to have found the association between a higher modMELD score and a higher risk of death, cerebral event, or pump thrombosis in patients with end-stage HF undergoing an LVAD implantation during a 1.5-year follow-up. The modMELD score has excellent prognostic power, as well as high sensitivity and specificity, allowing for a successful selection of patients with worse clinical outcomes during a 1.5-year follow-up.

The Model for End-stage Liver Disease (MELD) scoring system is commonly used as a prognostic tool in patients after a transjugular intrahepatic portosystemic shunt procedure [13]. The classic MELD score is calculated on the basis of serum bilirubin and serum creatinine levels as well as international normalized ratio value. However, patients with end-stage HF after an LVAD implantation require treatment with oral anticoagulants, and the usefulness of the classical MELD score may be limited. In order to exclude the impact of oral anticoagulation on INR, we used a modified version of the classical MELD scale—the modMELD score—replacing INR with albumin levels. This scoring system is a more reliable marker of risk in patients with elevated INR secondary to anticoagulation and may offer improved prognostic efficacy in patients undergoing an LVAD implantation. As indicated in several reports, the assessment of liver and kidney dysfunction according to the MELD and its modifications provides important information regarding the outcomes in patients with end-stage HF undergoing an LVAD implantation [14,15,16]. Critsinelis et al. showed that a higher MELD-XI score (MELD without INR) was associated with a lower postoperative survival rate and an increased risk of early right HF and infections compared with a lower MELD-XI score in patients with end-stage HF undergoing a continuous-flow LVAD implantation [14]. In turn, Bonde et al. demonstrated that a rising preoperative MELD score was a significant independent predictor for respiratory dysfunction, renal dysfunction, and mortality at six months after a VAD implantation [15]. Another study also showed that an LVAD implantation in patients with INTERMACS profile 2 or 1 and an increasing MELD score is associated with a high risk of death during a one-year follow-up [16]. 

Furthermore, we have observed a significant improvement in all parameters of liver and kidney function in patients after 6 months from the LVAD implantation. Yunus et al. also found that patients after an LVAD implantation who survived the first year showed an excellent recovery of their liver markers [17]. Other studies also showed an improvement in estimated glomerular filtration rate and other measures of kidney function over the first 3–6 months after an LVAD implantation [18,19,20]. In turn, Yoshioka et al. demonstrated that continuous-flow LVADs improve renal and hepatic function in patients with advanced HF, although in most patients, the initial improvement in renal function is largely transient, and the function returns to baseline after a prolonged support period [21]. Many patients with advanced HF before an LVAD implantation have symptoms of hepatopathy systemic hypoperfusion and passive congestion of the liver, which results from increased systemic venous pressure [17,22]. Similarly, kidney dysfunction secondary to HF also develops in response to chronically elevated central venous pressures, and the transmission of venous congestion to the renal veins impairs the glomerular filtration rate. [23,24]. In addition, a decrease in cardiac output in end-stage HF results in poor kidney perfusion, reduced renal autoregulation, increased renin–angiotensin system activation, and renal arterial vasoconstriction, which consequently further leads to kidney dysfunction [25]. These negative changes in peripheral organs secondary to HF can be reversed after the LVAD implantation. The LVAD therapy provides adequate perfusion to maintain normal end-organ function and may facilitate the recovery of liver and kidney dysfunction in patients with end-stage HF.

Another interesting finding of the present study is the association between higher TOS and OSI levels, as well as lower TAC levels, and the worse clinical outcomes in patients after the LVAD implantation. Among the analyzed oxidative stress parameters, OSI has the highest strength, sensitivity, and specificity to predict a composite endpoint in patients after an LVAD implantation during a 1.5-year follow-up.

Oxidative stress is essentially an imbalance between an increase in the formation of reactive oxygen species and the cells’ ability to remove or neutralize them by antioxidant systems [26]. Under conditions of prolonged exposure of cells to free radicals, antioxidant mechanisms are gradually depleted and play a crucial role in the initiation of cell damage and the following cascade of changes [26]. The deleterious effects of reactive oxygen species (ROS) are mainly due to the ability of ROS to produce changes in subcellular organelles and induce intracellular Ca_2_, modulating intracellular enzyme activity. ROS also have the ability to interact with proteins, lipids, and the genetic material of the nucleus and mitochondria, contributing to a change in their conformation, cell membrane integration, and, as a consequence, the impairment of normal cell function [26,27,28,29]. Those adverse effects of oxidative stress in the heart cause myocyte hypertrophy and contractile dysfunction, premature apoptosis, interstitial fibrosis, as well as endothelial vascular dysfunction [27,29]. Previous studies have demonstrated that ROS are associated with the development and progression of HF [29,30]. Increasing oxidative stress, along with a gradual decrease in the activity of antioxidant enzymes, leads to a disturbance of the oxidative–antioxidant balance towards the prooxidative state, which leads to the deterioration of heart function [26,27,28,29,30,31]. Our study has shown that patients with initially higher levels of oxidative stress identified as higher TOS and OSI levels, as well as lower TAC levels, have worse clinical outcomes after an LVAD implantation in a 1.5-year follow-up, despite comparable oxidative stress parameters after 6 months in both analyzed groups. The study by Caruso showed that an efficient pre-implant anti-oxidant system is associated with improved survival of the patients after an LVAD implantation [31]. However, the same authors also demonstrated that LV unloading and improved multi-organ function after 1 month from the LVAD implantation were not associated with a decrease in the oxidative stress markers serum levels [31]. It seems that a properly functioning e anti-oxidant system before surgery may play a protective role against vascular and organ damage in the perioperative and postoperative period, thus preventing adverse events [30]. In turn, an oxidative–antioxidative imbalance towards prooxidation may promote the development of multiple organ dysfunction in patients with HF [29,30,32]. That may explain worse clinical outcomes in patients after an LVAD implantation with initially worse parameters of oxidative stress.

The study has some limitations. Firstly, it is a single-center study with a relatively small number of patients undergoing an LVAD implantation. Further larger studies with validation samples are needed to assess the ability of the modMELD score and oxidative stress parameters to predict the clinical outcomes in patients after an LVAD implantation. The clinical utility of the presented results requires confirmation in larger, multicenter and prospective studies.

## 5. Conclusions

Our findings suggest that a modMELD score above the cut-off value is associated with a higher risk of death, cerebral event, and pump thrombosis within a 1.5-year follow-up. The modMELD score has excellent prognostic power, as well as high sensitivity and specificity, allowing for a successful selection of patients with worse clinical outcomes within a 1.5-year follow-up. The modMELD scoring system may be a useful predictor of worse outcomes in patients with end-stage HF undergoing an LVAD implantation. Furthermore, our study showed that higher TOS and OSI levels, as well as lower TAC levels, are associated with worse clinical outcomes in patients after an LVAD implantation. The holistic assessment of oxidative stress by OSI with excellent discriminatory power as well as acceptable sensitivity and specificity identifies patients with a higher risk of death, cerebral event, and pump thrombosis after an LVAD implantation within a 1.5-year follow-up.

## Figures and Tables

**Table 1:** Baseline characteristics of the patients before LVAD implantation.

Parameters	General Population N = 36 ^a^	Without MACE ^1^ N = 19	With MACE N = 17	*p* ^b^
Baseline data				
Age, years	58.0 (50.0–63.0)	58.0 (39.0–64.0)	58.0 (52.0–61.0)	0.8005
Male, n (%)	33 (86.8)	18 (94.7)	15 (88.2)	0.4811
Follow up, days	547.00 (522.5–547.0)	547.0 (547.0–547.0)	522.0 (515.0–533.0)	<0.0001 ^b^
Ischemic etiology, %	24 (66.7)	14 (73.7)	10 (58.8)	0.034 ^b^
BMI, kg/m^2^	27.4 (5.9)	27.1 (5.8)	27.6 (6.1)	0.7982
HR, bpm	71.31 (7.30)	70.74 (7.11)	71.94 (7.68)	0.628
SBP, mmHg	96.50 (86.50–100.00)	97.00 (89.00–100.00)	96.00 (84.00–100.00)	1
DBP, mmHg	66.25 (12.50)	66.05 (10.72)	66.47 (14.58)	0.9219
Comorbidities				
Hypertension, n (%)	16 (44.4)	8 (42.1)	8 (47.1)	0.7652
Type 2 diabetes, n (%)	16 (44.4)	7 (36.8)	9 (52.9)	0.3318
COPD, n (%)	2 (0.06)	1 (5.3)	1 (5.9)	0.9355
Persistent FA, n (%)	14 (38.9)	5 (26.3)	9 (52.9)	0.1018
Hypercholesterolemia, n (%)	17 (47.2)	8 (42.1)	9 (52.9)	0.5156
Pulmonary hypertension, n (%)	35 (97.2)	18 (94.7)	17 (100)	0.3374
Laboratory parameters				
WBC, ×10^9^/l	8.7 (6.7–9.9)	7.7 (6.7–10.3)	8.8 (6.7–9.7)	0.875
Hemoglobin, mmol/L	7.86 (1.2)	7.75 (1.2)	7.98 (1.2)	0.586
Platelets, ×10^9^/l	187.0 (134.0–268.5)	212.0 (164.0–274.0)	168.0 (125.0–238.0)	0.1294
Albumin, g/L	35 (32–39)	38 (34–41)	33 (31–38)	0.0714
Total protein, g/L	64.6 (7.9)	65.11 (8.3)	64.06 (7.8)	0.7009
ALT, U/I	33.5 (21.0–64.5)	34.0 (21.0–44.0)	33.0 (19.0–408.0)	0.6598
AST, U/I	37.0 (25.0–58.5)	31.0 (23.0–42.0)	45.0 (37.0–273.0)	0.0286 ^b^
Total bilirubin, µmol/L	24.8 (17.3–35.3)	18.5 (12.5–29.8)	33.5 (24.8–42.8)	0.0041 ^b^
hs-CRP, mg/L	8.0 (4.5)	6.9 (3.8)	9.3 (5.0)	0.1178
Creatinine, µmol/L	144.5 (44.4)	121.5 (31.6)	170.3 (43.1)	0.0004 ^b^
LDH, U/l	322. 5 (242.0–458.0)	289.0 (238.0–420.0)	430.0 (250.0–521.0)	0.1412
Urea, µmol/L	10.9 (7.3–14.6)	10.3 (6.9–14.1)	11.0 (9.2–18.0)	0.4066
NT-proBNP, pg/mL	9440 (6826–17,466.5)	7100 (3202–20,555)	(9220–16,531)	0.0722
Cholesterol, mmol/L	3.4 (2.9–4.5)	3.4 (3.1–3.9)	3.3 (2.3–4.9)	0.4067
LDL, mmol/L	1.7 (1.2–2.3)	1.9 (1.2–2.5)	1.50 (1.2–2.1)	0.3807
GGTP, U/I	141.5 (74.5–186.5)	140.0 (69.0–184.0)	154.0 (116.0–189.0)	0.7532
ALP, U/I	117.5 (59.0–159.0)	94.0 (54.0–136.0)	145.0 (75.0–174.0)	0.0485 ^b^
Glucose, mmol/L	5.5 (5.2–6.1)	5.3 (5.1–5.9)	5.6 (5.4–7.1)	0.281
Sodium, mmol/L	136.1 (3.6)	135.6 (3.1)	136.5 (4.1)	0.4612
Uric acid, µmol/L	466.9 (146.9)	479.1 (155.4)	453.4 (140.3)	0.6075
Fibrinogen, mg/dl	456.6 (104.2)	451.9 (95.9)	461.8 (115.5)	0.7811
TAC, mmol/L	1.3 (1.0–1.4)	1.4 (1.2–1.4)	1.1 (1.0–1.3)	0.0331 ^b^
TOS, μmol/L	2.9 (1.1)	2.3 (0.9)	3.8 (0.7)	<0.0001 ^b^
OSI	2.5 (1.7–2.8)	1.7 (1.3–2.2)	2.7 (2.6–4.5)	<0.0001 ^b^
ModMELD	17.2 (5.4)	14.01 (4.3)	20.7 (4.1)	<0.0001 ^b^
Echocardiographic parameters				
RVDD, mm	42.5 (40.5–45.0)	42.0 (41.0- 44.0)	45.0 (40.0–46.0)	0.2938
RVSP, mmHg	45.2 (12.4)	41.4 (10.6)	49.41 (13.3)	0.0512
TAPSE, mm	14.0 (12.0–16.0)	15.0 (12.0–16.0)	13.0 (12.0–17.0)	0.7865
LVEDD, mm	74.5 (68.0–81.5)	73.0 (67.0–83.0)	75.0 (69.0–80.0)	0.9372
LA, mmol/L	55.5 (7.5)	50.1 (9.3)	52.9 (6.7)	0.3177
LVEF, %	15.0 (11.0–15.5)	15.0 (10.0–18.0)	14.0 (12.0–15.0)	0.3978
Pharmacology treatment				
B-blockers, n (%)	35 (97.2)	18 (94.7)	17 (100)	0.3374
ACEI/ARB, n (%)	27 (75)	13 (68.4)	14 (82.4)	0.3352
Loop diuretics, n (%)	29 (80.6)	15 (78.9)	14 (82.4)	0.7966
MRA, n (%)	34 (94.4)	17 (89.5)	17 (100)	0.1687
Digoxin, n (%)	2 (0.06)	1 (5.3)	1 (5.9)	0.9355
Statin, n (%)	11 (30.6)	6 (31.6)	5 (29.4)	0.8879
Coumarin derivatives, n (%)	36 (100)	19 (100)	17 (100)	
Acetylsalicylic acid, n (%)	16 (44.4)	9 (47.4)	7 (41.2)	0.709
Clopidogrel, n (%)	20.0 (55.6)	10 (52.6)	10 (58.8)	0.709
Sildenafil, n (%)	35 (97.2)	18 (94.7)	17 (100)	0.3374
ICD, n (%)	24 (66.7)	13 (68.4)	11 (64.7)	0.8134
CRT-D, n (%)	12 (33.3)	6 (31.6)	6 (35.3)	0.8134

^a^ Data are presented as medians (upper and lower quartiles)), means (standard deviation), or numbers (percentage) of patients. ^b^ *p* < 0.05 (statistically significant). ^1^ MACE major adverse cardiac event-death, stroke, or pump thrombosis. Abbreviations: ACEI, angiotensin-converting-enzyme inhibitor; ALP, alkaline phosphatase; ALT, alanine aminotransferase; ARB, angiotensin II receptor blocker; AST, aspartate aminotransferase; BMI, body mass index; COPD, chronic obstructive pulmonary disease; CRT-D, cardiac resynchronization therapy—defibrillator; DBP, diastolic blood pressure; FA, atrial fibrillation; GGTP, gamma-glutamyl transpeptidase; HR, heart rate; hs-CRP, high-sensitivity C-reactive protein; ICD, implantable cardioverter-defibrillator; LA, left atrium; LDH, Lactate dehydrogenase; LDL, low-density lipoprotein; LVEDd, left ventricular end-diastolic dimension; LVEF, left ventricular ejection fraction; modMELD, modified Model for End-Stage Liver Disease excluding INR; MRA, mineralocorticoid receptor antagonists; NT-proBNP, N-terminal prohormone of brain natriuretic peptide; OSI, oxidative stress index; RVDD, right ventricular end-diastolic dimension; RVSP, right ventricular systolic pressure; SBP, systolic blood pressure; TAC, total antioxidant capacity; TAPSE, tricuspid annular plane systolic excursion; TOS, total oxidant status; WBC, white blood cells.

**Table 2:** Laboratory parameters before and 6 months after LVAD implantation.

	Before LVAD Implantation N = 36 ^a^	6 Months after LVAD Implantation N = 36	*p* ^b^
Albumin, g/L	35.2 (5.8)	45.1 (3.7)	<0.0001 ^b^
Total protein, g/L	64.6 (7.9)	75.4 (4.4)	<0.0001 ^b^
ALT, U/I	33.5 (21.0–64.5)	17.0 (13.5–24.0)	<0.0001 ^b^
AST, U/I	37.0 (25.0–58.5)	21.5 (17.0–28.0)	<0.0001 ^b^
GGTP, U/I	141.5 (74.5–186.5)	47.5 (33.0–109.5)	<0.0001 ^b^
ALP, U/I	117.5 (59.0–159.0)	95.5 (72.5–118.5)	0.2193
Total bilirubin, µmol/L	24.8 (17.4–35.3)	11.1 (7.3–14.2)	<0.0001 ^b^
hs-CRP, mg/L	7.0 (4.4–11.4)	3.4 (2.3–7.0)	0.0008 ^b^
Creatinine, µmol/L	137.5 (109.5–183.5)	102.5 (95.5–127.5)	<0.0001 ^b^
NT-proBNP, pg/mL	9440.0 (6826.0–17,466.5)	1347.0 (764.7–3299.5)	<0.0001 ^b^
Na, mmol/L	136.1 (3.6)	139.5 (2.0)	<0.0001 ^b^
Uric acid, µmol/L	466.9 (146.9)	456.5 (114.3)	0.7
Fibrinogen, mg/dl	449.5 (368.5–522.0)	389.0 (343.5–441.0)	0.0191 ^b^
TAC, mmol/L	1.3 (1.0–1.4)	1.2 (1.1–1.4)	0.6165
TOS, μmol/L	3.1 (2.1–3.8)	2.2 (1.3–4.4)	0.4776
OSI	2.5 (1.7–2.8)	1.9 (1.0–3.9)	0.7118
modMELD	16.5 (13.0–21.7)	7.9 (7.3–0.1)	<0.0001 ^b^

^a^ Data are presented as medians (upper and lower quartiles), means (standard deviation), or numbers (percentage) of patients. ^b^ *p* < 0.05 (statistically significant). Abbreviations: see Table 1.

**Table 3:** A summary of the ROC curves analysis of biomarkers predicting composite end-point during 1.5-years follow-up.

	AUC (±95 CI)	*p*	Cut-Off	Sens. (±95 CI)	Spec. (±95 CI)	PPV	NPV	Accuracy
TAC	0.7183 (0.5417–0.8948)	<0.01	<1.37	0.99 (0.80–0.99)	0.42 (0.20–0.67)	0.61 (0.41–0.79)	0.99 (0.64–0.99)	0.69 (0.52–0.84)
TOS	0.9149 (0.8205–0.9900)	<0.01	>3.28	0.82 (0.57–0.96)	0.89 (0.67–0.99)	0.88 (0.63–0.98)	0.85 (0.62–0.97)	0.86 (0.62–0.97)
OSI	0.9628 (0.9030–0.9878)	<0.01	>2.48	0.99 (0.80–0.99)	0.89 (0.67–0.99)	0.89 (0.67–0.99)	0.99 (0.80–0.99)	0.94 (0.81–0.99)
modMELD	0.8700 (0.7494–0.9905)	<0.01	>17.55	0.82 (0.57–0.96)	0.84 (0.60–0.97)	0.82 (0.57–0.96)	0.84 (0.60–0.97)	0.83 (0.60–0.94)

Abbreviations: see Table 1.

## Data Availability

The data presented in this study are available on request from the corresponding author. The data are not publicly available due to privacy restrictions related to the rules in our institution.
